# Single-dose intramuscular methotrexate for treatment of cervical ectopic pregnancy: A case report

**DOI:** 10.1016/j.crwh.2021.e00340

**Published:** 2021-07-08

**Authors:** Elizabeth Dilday, Christopher Douglas, Kathleen Brennan

**Affiliations:** University of California, Los Angeles Department of Obstetrics and Gynecology, Division of Reproductive Endocrinology and Infertility, David Geffen School of Medicine, Los Angeles, California, 10833 Le Conte Ave CHS 27-139, Los Angeles, CA 90095, USA

**Keywords:** Cervical ectopic pregnancy, Methotrexate, Medical management, In vitro fertilization, Reproductive endocrinology, Infertility

## Abstract

**Introduction:**

Cervical ectopic pregnancy (CEP) is a rare but potentially life-threatening phenomenon, and conclusive management guidelines have not been elucidated. Patients undergoing assisted reproductive technologies (ART) are at increased risk of CEP and noninvasive, fertility-sparing treatments are necessary for this population. This case report demonstrates the safety and efficacy of a single dose of intramuscular methotrexate for CEP in early gestation.

**Case Description:**

A 45-year-old patient (G3P0030) presenting with painless vaginal bleeding was found to have CEP on transvaginal ultrasound at 5 weeks and 1 day of gestation after undergoing day-5 frozen embryo transfer. She was given one 50 mg/m^2^ dose of intramuscular methotrexate and she remained in a stable condition while being observed in the hospital. Her beta-hCG level decreased 38.2% between day 4 and day 7 after treatment and returned to nonpregnancy levels by day 28.

**Discussion:**

A single dose of intramuscular methotrexate is an effective, noninvasive, fertility-sparing method of treatment for CEP in patients who are early in gestation and hemodynamically stable. This is a recommended option, especially for those undergoing fertility treatment. Further studies need to be performed to formulate national guidelines regarding the treatment of CEP.

## Highlights

• Cervical ectopic pregnancy (CEP) is rare and potentially life-threatening.
• Use of assisted reproductive technologies (ART) increases patients' risk of CEP.
• Most patients with CEP after the use of ART desire fertility-sparing treatment when possible.
• In the case described, single-dose methotrexate was used as a fertility-sparing treatment of CEP.

## 1 Introduction

Cervical ectopic pregnancy (CEP) comprises 0.1–1% of all ectopic pregnancies and occurs in 1 in 10,000 to 1 in 18,000 of all pregnancies [1,2]. Additionally, 3.7% of all in vitro fertilization (IVF) ectopic gestations occur in the cervix [2], and several reports suggest an increase in CEP in women undergoing IVF [2,3]. The mechanism of cervical gestation after IVF is poorly understood, but it may be related to contractions of the junctional zone of the uterus in the luteal phase as a consequence of progesterone elevation [3].

In addition to assisted reproductive technologies (ART), potential risk factors for this rare condition include prior dilation and curettage (D&C), spontaneous abortion, cesarean delivery, and use of intrauterine devices [2]. D&C may put patients at risk for CEP due to damage of the endometrial lining preventing implantation of the fertilized ovum in the uterus. Inflammatory conditions (such as endometritis and pelvic inflammatory disease), mechanical risk factors (such as fibroids), congenital uterine malformations, and alterations in uterine tone can also contribute to the development of CEP [1].

The most common symptom of CEP is painless vaginal bleeding, and one-third of affected patients present with massive hemorrhage [1]. Clinical criteria to aid in diagnosis of CEP include painless bleeding after amenorrhea, a softened and disproportionately enlarged cervix equal to or larger than the uterine body, products of conception entirely confined within, and firmly attached to, the endocervix, and a “snug” internal os or partially opened external os [1]. Six sonographic diagnostic criteria for CEP are: (1) cervical enlargement, (2) uterine enlargement, (3) diffuse amorphous intrauterine echoes, (4) absence of intrauterine pregnancy (IUP), (5) peritrophoblastic blood flow to the conceptus by color flow Doppler (a nonviable sac does not have this blood flow), and (6) absence of the “sliding sign,” whereby the gestational sac of an abortus slides against the endocervical canal after gentle pressure on the cervix with a transvaginal probe [4,5].

Treatment options for CEP involve medical, surgical or expectant management. In a review that analyzed 252 cases, 37.6% of patients received a combination of medical and surgical treatment, 34.9% underwent medical treatment and 27.5% underwent surgical treatment [1]. Surgical management options include D&C, hysterectomy and surgical aspiration. Hysterectomy may be considered the first-line treatment if CEP is diagnosed in the second trimester and if the patient is hemodynamically unstable, is a Jehovah's witness, or if she has completed childbearing [1]. CEP may be amenable to conservative management if it is identified before 12 weeks, if the pregnancy is without fetal cardiac activity, or if the serum hCG level is low. Medical management of CEP uses systemic methotrexate, administered in a single-dose or multi-dose fashion, similar to regimens used for tubal ectopic pregnancy [6]. Medical management of CEP with methotrexate carries an 11% risk of major hemorrhage and a 3% risk of hysterectomy, compared with a 35% risk of major hemorrhage and 15% risk of hysterectomy with surgical management [3].

To our knowledge, there are only twelve reported cases of exclusive CEP after IVF [3]. The other case reports describe CEP in the setting of heterotopic pregnancy. Because CEP is so rare, there is an absence of conclusive treatment guidelines. Thoughtful management strategies are especially warranted in the setting of ART, in which patients highly desire future fertility. This report describes a cervical ectopic pregnancy following embryo transfer, managed successfully with intramuscular methotrexate therapy alone. Informed consent was obtained from the patient for publication of this report.

## 2 Case Presentation

A 45-year-old patient (G3P0030) desired pregnancy and sought fertility treatment in an academic reproductive endocrinology and infertility center. Her medical history included hypertension, prediabetes, thyroid dysfunction and overweight habitus (body mass index of 29 kg/m^2^). Her medications included methyldopa, metformin and levothyroxine. Her past surgeries included laparoscopic proximal transection of the left fallopian tube for left hydrosalpinx with concurrent loop electrode excisional procedure (LEEP) for cervical intraepithelial neoplasia (CIN) II at age 41. Pathology was negative for dysplasia and malignancy. She had previously undergone three hysteroscopies with endometrial polypectomies.

Her obstetrical history included failed fertility treatments and recurrent pregnancy loss. Her first pregnancy, conceived naturally five years earlier, ended in spontaneous abortion at 6 weeks of gestation. She underwent three cycles of IVF and had one fresh day 5 transfer of three embryos that did not result in pregnancy. She then had three separate programmed frozen embryo transfer cycles using autologous oocytes. In two cycles, two embryos were transferred, resulting in biochemical pregnancies. In the third cycle, three embryos were transferred, resulting in a negative pregnancy test. She later underwent two frozen embryo transfer cycles of donor ovum-derived embryos. She had one embryo transferred, resulting in a negative pregnancy test, and another single embryo transfer the following cycle, ending in a first-trimester spontaneous abortion. Workup for recurrent pregnancy loss was unremarkable. She had a normal female karyotype, negative antiphospholipid antibody testing and negative anti-thyroglobulin and anti-thyroid peroxidase antibodies. Her partner had a normal male karyotype.

Next, she underwent a programmed transfer of one donor ovum-derived day 5 frozen euploid embryo. Nine days later, she underwent initial beta-hCG testing, which reported a level of 53 mIU/mL. Two days later, beta-hCG had increased to 155 mIU/mL. She reported minimal vaginal spotting at that time. Four days later, at 4 weeks and 6 days of gestation, she paged the on-call physician to report abdominal cramping along with brown spotting. Pain resolved later that day but vaginal spotting continued. The following day, at 5 weeks and 0 days of gestation, her beta-hCG level continued to rise, to 2176 mIU/mL.

**Fig. 1:**
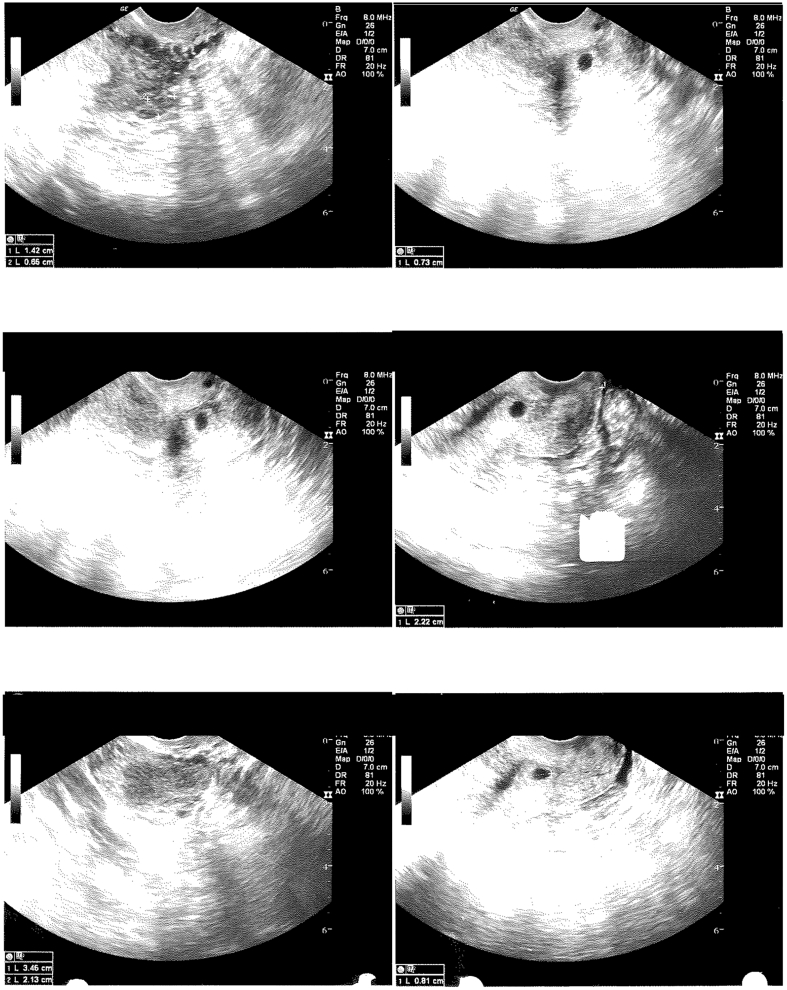
Transvaginal ultrasound at 5 weeks and 1 day of gestation. Gestational sac measuring 8.1 mm in the lower uterine segment versus the upper cervix. Gestational sac measuring 2.2 cm from the distal end of the cervix. No yolk sac or fetal pole visualized. Beta-hCG 3217 mIU/mL.

**Fig. 2:**
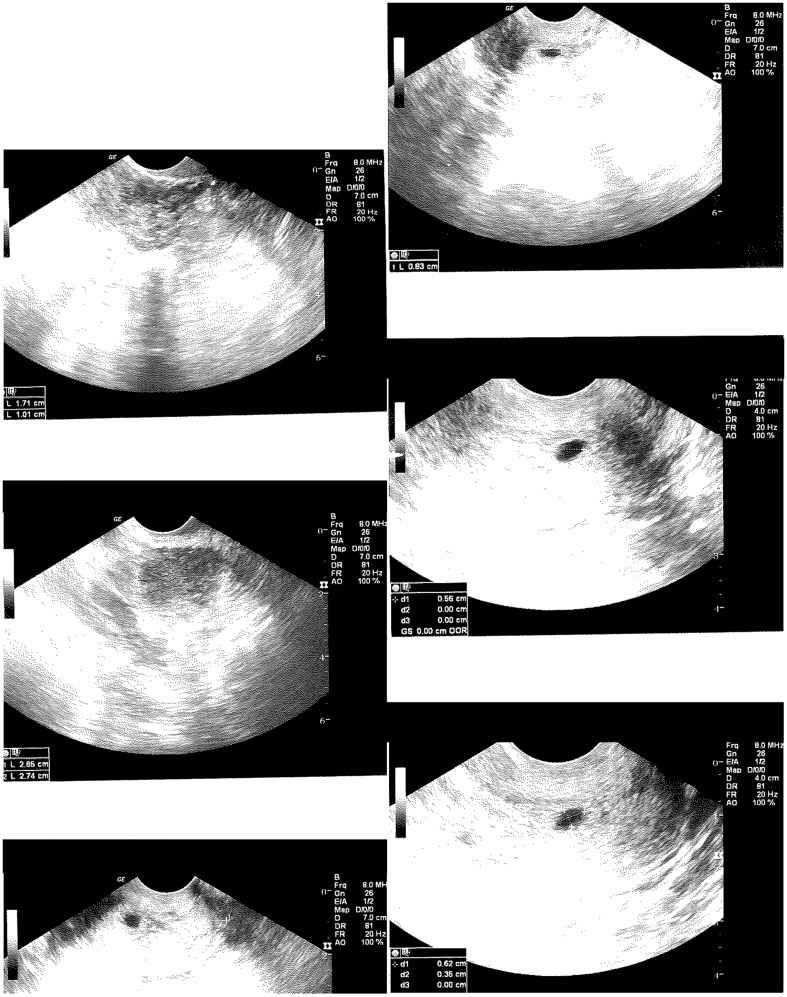
Transvaginal ultrasound at 5 weeks and 2 days of gestation. Gestational sac measuring 8.3 mm in the upper cervix. No yolk sac or fetal pole visualized. Beta-hCG 4607 mIU/mL.

Her first transvaginal ultrasound was the following day, at a gestational age of 5 weeks and 1 day (Fig. 1). Transvaginal ultrasound imaging showed a gestational sac measuring 8.1 mm in the lower uterine segment versus the upper cervix. The sac was located 2.2 cm from the external cervical os. A yolk sac and fetal pole were not visualized. Beta-hCG was noted to be 3217 mIU/mL on this day. Transvaginal ultrasound was repeated the following day, given concern for CEP (Fig. 2). The gestational sac was in the upper cervix, measuring 8.3 mm. Again, no yolk sac or fetal pole was seen. Her beta-hCG level increased to 4607 mIU/mL on this day. Intramuscular methotrexate was then recommended for likely CEP. The patient refused methotrexate at that time as the pregnancy was highly desired, and the patient was discharged with strict return precautions.

Later that day, the patient reported heavier vaginal bleeding at home with passage of large clots. On arrival at the emergency room, she reported soaking through two pads every 30 minutes. She was then amenable to methotrexate treatment given heavy vaginal bleeding and she was given a single-dose intramuscular methotrexate protocol (50 mg/m^2^). She was admitted for hemodynamic monitoring and observation.

Overnight, she had approximately 400–500 cc of vaginal bleeding. Her vital signs remained stable throughout admission. Her hemoglobin level decreased from 12.0 g/dL at baseline prior to admission to 8.9 g/dL on the morning of hospital day 2 (HD), then to 8.5 g/dL on the evening of HD 2. By HD 3, vaginal bleeding was minimal and pain had resolved. Her hemoglobin level stabilized at 8.3 g/dL on the day of discharge. She was discharged with oral iron supplementation and plan for serial beta-hCG testing, with activity restrictions and strict return precautions.

**Fig. 3:**
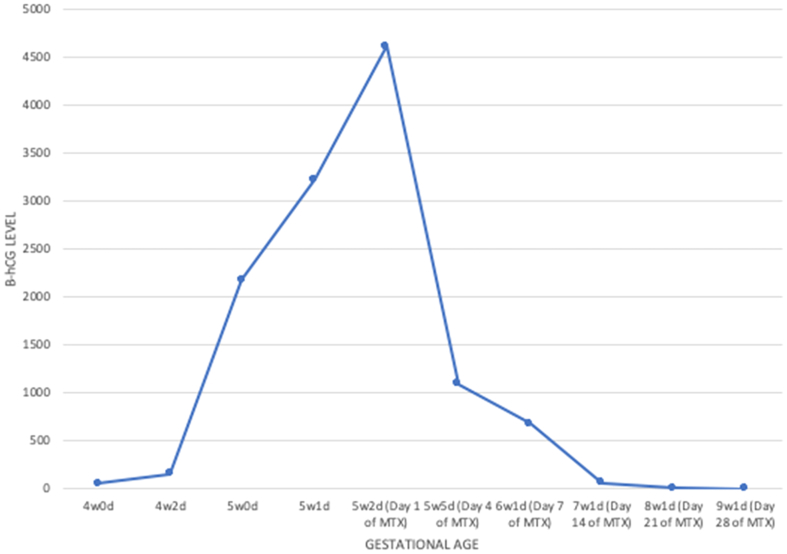
Beta-hCG trend. There was a decrease of 38.2% between day 4 and day 7 after one dose of intramuscular methotrexate.

Between days 4 and 7 of methotrexate treatment, the beta-hCG decreased by 38.2% (Fig. 3). By day 7, she reported minimal spotting and no pain. Beta-hCG continued to downtrend weekly thereafter (Fig. 3). She did not require any additional methotrexate doses or procedures to facilitate resolution of the pregnancy.

Three months after the final beta-hCG measurement, she had a follow-up saline sonohysterogram that showed a normal endometrial cavity.

## 3 Discussion

Maternal mortality with CEP is as high as 6% [1]. The development and application of safe and effective treatment strategies are paramount given this risk. This report demonstrates that a single intramuscular dose of methotrexate was a safe, effective and noninvasive treatment for CEP. Medical management should be limited to patients who are hemodynamically stable [6]. According to the American College of Obstetricians and Gynecologists (ACOG), absolute contraindications to methotrexate use include intrauterine pregnancy, blood dyscrasias, active breastfeeding, as well as pulmonary, hepatic, renal or peptic ulcer disease [6]. ACOG also recommends relative contraindications to methotrexate administration, such as a high initial beta-hCG level greater than 5000 mIU/mL, given the higher risk of treatment failure [6]. The failure rate of methotrexate therapy is 14.3% or higher with a pretreatment hCG level greater than 5000 mIU/mL, compared with a 3.7% failure rate for beta-hCG levels less than 5000 mIU/mL [6]. This patient's beta-hCG at the time of diagnosis was 3217 mIU/mL and peaked at 4607 mIU/mL.

In this case, there was early diagnosis of the ectopic pregnancy at its unusual location because of her close monitoring. Additionally, she had known risk factors for CEP, including history of ART, LEEP, and three hysteroscopic polypectomies. Patients attempting conception via ART often undergo close prospective monitoring, which enables prompt diagnosis and facilitates timely intervention in abnormal pregnancies. Identification of CEP after the 10th week of gestation carries greater risk of hemorrhage, hypovolemic shock and need for emergency hysterectomy [3].

Methotrexate treatment requires close monitoring of serum beta-hCG levels to ensure adequate response. Patients must be willing to adhere to close follow-up requirements prior to considering this method of treatment. With the single-dose regimen of methotrexate, the target beta-hCG decrease between day 4 and day 7 is 15% [6]. An additional dose of methotrexate is advised if the decline is less than 15%. In this case, the patient's serum beta-hCG level had decreased by 38.2% in that time period. Recent data demonstrates improved efficacy with two doses of methotrexate, including statistically significantly greater success with high hCG levels and shorter length of follow-up to treatment success by 7.9 days [7]. The two-dose protocol also showed lower odds of surgery that was not statistically significant [7]. We now use this protocol routinely and feel it is a safe and effective treatment for CEP.

This case is unique given early identification of the abnormality and successful treatment with a single intramuscular dose of methotrexate. There are also reports of intracervical injection of methotrexate for CEP [8]. However, this route has been shown to be associated with a high risk of bleeding [1]. Methotrexate has also been combined with other treatment modalities for CEP. There are two cases of treatment of first trimester cervical ectopic pregnancy with single-dose systemic methotrexate followed by D&C [9]. Another case describes a single intramuscular dose of methotrexate and transvaginal ultrasound-guided aspiration with a 16-gauge ovum aspiration catheter [10]. However, this patient's course was complicated by bleeding and she required transfusion of 2 units packed red blood cells following therapy. Two intramuscular doses of methotrexate were combined with bilateral uterine artery embolization in another case diagnosed at 8 weeks of gestation [11]. One case series investigated patients treated with a combination of intra-arterial methotrexate infusion and uterine artery embolization (UAE) and showed that this group had quick downtrend of serum beta-hCG, rapid cervical mass elimination and no severe complications compared with patients treated with systemic intramuscular methotrexate or uterine artery embolization alone [12]. Another report has described the combination of uterine artery embolization and intramuscular methotrexate as late as 12 weeks 3 days, but the patient had significant postoperative complications requiring admission to the intensive care unit for sepsis and acute kidney injury [13].

Several other medications, including potassium chloride, prostaglandins, hydrogen peroxide and mifepristone, have shown efficacy in the treatment of CEP [1]. Local injection of potassium chloride under ultrasound guidance is an alternative to methotrexate therapy. Potassium chloride can be used alone, in combination with methotrexate, or in the setting of failed methotrexate treatment. There was a report of prostaglandin use both systemically and intra-amniotically for a CEP at 9 weeks of gestation. However, this case was complicated by poorly controlled hemorrhage requiring hysterectomy [14]. A subsequent case series discussed three cases of first trimester CEP successfully treated with local prostaglandin instillation (12.5–25 μg sulprostone) combined with curettage, with no report of postoperative hemorrhage [15]. One case series showed that hysteroscopy with intrauterine irrigation with 3.5% hydrogen peroxide was a safe and effective treatment [16]. A series of 11 CEP cases were treated with one dose of intramuscular methotrexate and two doses of oral mifepristone 25 mg, all given on the same day [17]. However, most of these cases required an additional curettage and two required a hysterectomy for poorly controlled hemorrhage. As described, a multitude of methods have been tried for the treatment of CEP. Further study is warranted to elucidate the most effective treatment for patients who are potentially not candidates for methotrexate treatment or certain types of surgical management.

Given that patients undergoing fertility treatment are at an increased risk of CEP, noninvasive treatments that can preserve fertility are imperative. While there has been no consensus on the best treatment [3], this case demonstrates that a single intramuscular dose of methotrexate alone is safe and effective. This is the first case report demonstrating successful treatment of CEP with a single dose of methotrexate in a patient undergoing ART. We recommend this approach for patients undergoing fertility treatment who present with CEP early in gestation and are hemodynamically stable.
